# Association between Stigma and Pain in Patients with Temporomandibular Disorders

**DOI:** 10.1155/2022/2803540

**Published:** 2022-09-21

**Authors:** Rui Zhu, Li Zhang, Yun-Hao Zheng, Zi-Han Zhang, Li-Ming Zhang, Hao-Lun Yang, Yuan Yue, Xin Xiong

**Affiliations:** ^1^The State Key Laboratory of Oral Diseases and National Clinical Research Center for Oral Diseases, Department of Prosthodontics, West China Hospital of Stomatology, Sichuan University, Sichuan, China; ^2^The State Key Laboratory of Oral Diseases and National Clinical Research Center for Oral Diseases, Department of Orthodontics, West China Hospital of Stomatology, Sichuan University, Chengdu, Sichuan, China; ^3^Rehabilitation Medicine Center, Department of Rehabilitation Medicine, West China Hospital, Sichuan University, Chengdu, Sichuan, China; ^4^Department of Temporomandibular Joint, West China Hospital of Stomatology, Sichuan University, Chengdu, Sichuan, China

## Abstract

**Objective:**

This study aims to explore the association between stigma and pain in patients with temporomandibular disorders (TMDs).

**Methods:**

Two hundred and twenty-five patients with TMDs were recruited, and they completed the questionnaires including the Visual Analogue Scale of Pain (VAS), Generalized Anxiety Disorder 7-Item (GAD-7), the Patient Health Questionnaire 9-item (PHQ-9), Jaw Functional Limitation Scale 8-item (JFLS-8), the Stigma Scale for Chronic Illness 8-item (SSCI-8), and other demographic and disease-related information. The total score of SSCI-8 indicated overall stigma, which could be classified into 2 subdomains, felt stigma and enacted stigma, according to their representative items, respectively. Then, the patients were divided into 2 groups in each subdomain of stigma according to their scores: stigma group (score ≥ 1) and no stigma group (score = 0).

**Results:**

Patients with overall stigma and enacted stigma presented significantly higher scores in VAS, GAD-7, PHQ-9, and JFLS-8 than those without overall stigma and those without enacted stigma, respectively. Significant differences between patients with and without felt stigma were only observed in GAD-7, PHQ-9, and JFLS-8. Patients with overall stigma and enacted stigma mainly suffered from pain-related TMDs (PTs) and combined TMDs (CTs). Overall stigma and enacted stigma rather than felt stigma were significantly associated with both PTs and CTs. Stigma, including overall stigma, enacted stigma, and felt stigma, was more associated with anxiety and depression and less related to jaw functional limitation of the patients with TMDs.

**Conclusion:**

Stigma, specifically enacted stigma, was correlated to pain in patients with TMDs. Stigma was more related to psychological problems than jaw functional limitation.

## 1. Introduction

Temporomandibular disorders (TMDs) are a group of diseases affecting the masticatory muscles, temporomandibular joint (TMJ), and associated structures [1–3]. The overall prevalence of TMDs is approximately 31%, and the occurrence of painful TMDs varied from 3.4% to 12%, with 65% recurrent rate [4–7]. Pain in the TMJ, sounds of the joint, and disability of mouth opening are the most common symptoms of TMDs [7, 8] that affect people's mastication and sleeping, leading to a poor quality of life [9]. According to the Diagnostic Criteria for TMDs, TMDs can be classified into pain-related TMDs (PTs) and intra-articular TMDs (ITs) [10]. PTs contain various maxillofacial pains attributed to TMDs such as myalgia, headache, and others, and ITs include TMJ subluxation, disc displacements, and degenerative joint disease (DJD) [11]. Patients could suffer from multiple TMDs at the same time.

The etiologies of TMDs are multifactorial and complex, including environmental, biological, psychological, social, and cognitive factors [12]. Psychological conditions including stress, anxiety, and depression play a role in painful TMDs [13, 14]. Individuals with TMDs often feel stressed, anxious, and depressed in their daily life, and these unhealthy psychological conditions will in turn act as risk factors in the progression of painful TMDs [15]. Thus, it is quite important to focus on the mental health and psychological changes of the patients during the research and treatment of TMDs. However, the psychological conditions of the patients with TMDs are not only associated with the symptoms and severity of the disease themselves but also related to the awareness held by the patients and others, such as stigma.

Stigma was first defined as an “undesired differentness” that disqualifies individuals from full social acceptance by Goffman in 1963 [16]. It refers to the attitudes of avoidance, rejection, and fear as being different that keep patients away from receiving treatment [17]. Stigma can originate both internally and externally. Felt stigma (internal stigma) is the attitudes of the individual experiencing diseases, and enacted stigma (external stigma) refers to the opinions held by family members, friends, and others [18]. A survey reported that more than a third of the patients with chronic pain experienced stigma in which they felt alienated and socially withdrawn [19]. TMD patients, especially those suffering from chronic pain, more often experienced stigma when the TMD was attributed to psychological causes [20]. Their friends and family may consider the chronic pain exaggerated and regard it as a mental disease, which will induce the negative and depressed emotions of the patients [20, 21]. Nowadays, although stigma has been found in the TMD patients for years [17, 20], the association between stigma and TMD was rarely investigated.

In this study, we collected the sociodemographic and clinical data closely or potentially related to stigma, aiming to explore the relationship between stigma and pain in TMD patients and to provide theoretical basis for paying attention to patients' stigma during the treatment of TMD.

## 2. Materials and Methods

### 2.1. Study Design and TMD Diagnosis

This research was conducted in accordance with the Declaration of Helsinki and approved by the Ethics Committee of the West China School of Stomatology of Sichuan University.

A total of 225 patients with TMDs attending the Department of Temporomandibular Joint in the West China Hospital of Stomatology participated in the study. All the patients were volunteers, and the informed consent form was given before participation. The inclusion criteria were as follows: (a) patients attending our department and diagnosed as TMD for the first time and (b) patients aged 12 years or above. The exclusion criteria were as follows: (a) presence of major trauma and/or operations; (b) presence of drug abuse; (c) presence of non-TMD joint and/or muscle diseases; (d) current consumption of central nervous system agents; (e) cognitive impairment and/or illiteracy; and (f) incomplete or poorly completed questionnaire. Demographic information, including gender, age, and education level, and disease-related characteristics involving dental treatment histories, systemic diseases histories, adverse oral habits, the current score of Visual Analogue Scale of Pain (VAS current), the highest score of VAS in the last month (VAS highest), Generalized Anxiety Disorder 7-item (GAD-7), the Patient Health Questionnaire 9-item (PHQ-9), Jaw Functional Limitation Scale 8-item (JFLS-8), and the Stigma Scale for Chronic Illness 8-item (SSCI-8) were gathered. The TMD examination was conducted according to the DC/TMD protocol by an experienced specialist of TMD. The diagnosis of TMD was determined based on the DC/TMD “diagnostic tree” and associated algorithms. And in this research, TMD was further classified into only pain-related TMD (PT), only intra-articular TMD (IT), and combined TMD (CT), which embodied both PT and IT.

### 2.2. Measurements

#### 2.2.1. Stigma

The stigma among patients was evaluated using the Chinese version of the SSCI-8, demonstrating high reliability and validity [22]. The SSCI-8 contains 8 items for overall stigma with 2 subscales, felt stigma (2 items) and enacted stigma (6 items). It is a 5-point Likert scale from 1 (never) to 5 (always). The total scores of SSCI-8 range from 1 to 40, in which a higher score indicates a higher frequency of experiencing stigma.

In this study, stigma was comprehensively elucidated from 3 perspectives: overall stigma, felt stigma, and enacted stigma. Each stigma was analyzed in the same way. The scores of SSCI-8 no less than 1 were considered as experiencing stigma.

#### 2.2.2. Psychological Conditions

The patients' psychological conditions were assessed using PHQ-9 and GAD-7. PHQ-9 is a 9-item questionnaire focusing on depression. The total scores of PHQ-9 range from 0 to 27, in which the score is positively correlated to the degree of depression. GAD-7 assesses the frequency of general anxiety. It is a 4-point Likert scale from 1 (never) to 4 (nearly every day) with 7 items.

#### 2.2.3. Jaw Function Limitation

The patients' jaw function limitation was assessed using the JFLS-8 concerning jaw function (jaw mobility, mastication, and verbal and emotional expression). It is an 11-point scale (from no limitation to severe limitation) with 8 items.

#### 2.2.4. VAS

The pain intensity of the temporomandibular joint region including masticatory muscles was measured using the VAS. The VAS evaluates pain on a scale from 0 (no pain) to 10 (the maximum pain imaginable). In this study, we gathered the information about the current pain level and the most severe pain level in the last month, referring to the VAS current and the VAS highest, respectively.

### 2.3. Statistical Analysis

The sample size was calculated using *G*^*∗*^ Power software with *α* = 0.05 and power = 0.80. According to previous studies [23], the mean value for the psychological discomfort in the mild TMD group was 5.05 ± 5.63 compared with 8.08 ± 4.67 in the moderate TMD group. The sampling ratio (patients with stigma/patients without stigma) and standard deviation (SD) within each group were assumed as 0.4 and 6, respectively. The analysis demonstrated that 154 subjects were necessary in the study with an effect size of 0.51. The sample size of 225 was sufficient in this study.

Frequencies and percentages were used to describe gender distribution, education level, adverse oral habits, dental treatment, systemic diseases, PT, IT, and CT. Mean ± SD was used to present quantitative data such as age, VAS current, VAS highest, GAD-7, PHQ-9, and JFLS-8.

Spearman's correlation coefficients were calculated among overall stigma, felt stigma, enacted stigma, GAD-7, PHQ-9, and JFLS-8. And the Spearman's *r* values of 0.40, 0.60, and 0.80 were regarded as weak, moderate, and strong associations, respectively.

Univariate regression analysis was carried out to observe the crude association among variables. Multivariable linear regression analyses for PT, IT, and CT in overall stigma, felt stigma, and enacted stigma were performed after adjusting the confounders that were selected on the basis of their associations with the outcomes of interest or a change in effect estimate of more than 10%. The Hochberg method was used to adjust the *P* values of independent correlation analysis. The test level was set as *α* = 0.05, and adjusted *P* values <0.05 were considered statistically significant.

## 3. Results

### 3.1. Participants and General Results

Two hundred and twenty-five patients with TMDs were enrolled in this study (Table 1). The average age of the patients was 27.30 ± 8.35 years, ranging from 12 to 58. Of the 225 patients, 56 (24.89%) were male and 169 (75.11%) were female. The patients mostly graduated from university or junior college (68.00%), had adverse oral habits (78.67%), received dental treatment (74.67%), and did not have systemic diseases (85.33%).

Cronbach's *α* was used to assess the reliabilities of the scales. For SSCI-8, PHQ-9, GAD-7, and JFLS-8, *α* was 0.787, 0.903, 0.924, and 0.806, respectively.

### 3.2. Overall Stigma, Felt Stigma, and Enacted Stigma

Results (Table 2) revealed that the patients with overall stigma presented significantly higher scores in the VAS current, VAS highest, GAD-7, PHQ-9, and JFLS-8 than those without overall stigma. Patients with overall stigma mainly suffered from PT (*P*=0.025) and CT (*P*=0.004). Besides, the patients with overall stigma also presented worse oral habits (*P*=0.090).

Significant differences between patients with and without felt stigma were only observed in GAD-7, PHQ-9, and JFLS-8 (Table 3). The patients with felt stigma mainly experienced worse psychological and TMD problems than those without felt stigma. Moreover, patients with felt stigma possessed a lower level of education (*P*=0.071) than those without felt stigma.

The scores of the VAS current, VAS highest, GAD-7, PHQ-9, and JFLS-8 were significantly higher in patients with enacted stigma than those without enacted stigma. The age differences (*P*=0.05) between patients with enacted stigma (28.69 ± 9.83) and the patients without enacted stigma (26.45 ± 7.21) were too small to be clinically meaningful. Patients with enacted stigma mainly suffered from PT (*P*=0.013) and CT (*P*=0.006).

### 3.3. Correlations

**Figure 1:**
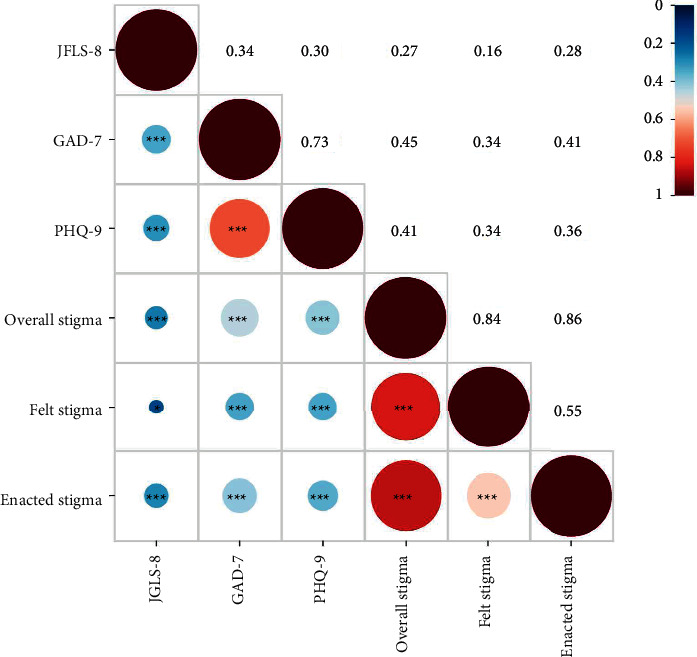
The heatmap of correlations among the study variables. Spearman's correlation analysis is used. ^*∗*^*P* value <0.05; ^*∗∗∗*^*P* value <0.001.

Correlations among the studied variables in Figure 1 showed that JFLS-8, GAD-7, and PHQ-9 were all positively correlated to overall stigma, felt stigma, and enacted stigma. Overall stigma showed moderate correlations with GAD-7 (*r* = 0.45, *P* < 0.05) and PHQ-9 (*r* = 0.41, *P* < 0.05). For felt stigma, the associations among JFLS-8, GAD-7, and PHQ-9 were weak (*r* = 0.16–0.34). A moderate correlation was found between enacted stigma and GAD-7 (*r* = 0.41, *P* < 0.05). Stigma was most associated with GAD-7, followed by PHQ-9 and JFLS-8. Effects of clinical characteristics and quantitative variables on enacted stigma are shown in Table 4. The univariable linear regression analysis shown in Table 5 revealed that overall stigma and enacted stigma rather than felt stigma were significantly associated with PT and CT. No significant associations among the variables were observed in IT. The results of the multivariable linear regression analysis (Table 6) indicated that, after adjusting the confounders, overall stigma and enacted stigma were still significantly associated with PT and CT, while felt stigma was not. Age was selected as the covariate in multivariable analysis model 1 in PT and CT. In model 1, the OR of overall stigma was 1.93 (95% CI 1.04 to 3.58, *P* < 0.05) in PT and 2.24 (95% CI 1.26 to 3.98, *P* < 0.05) in CT. The OR of enacted stigma was 2.09 (95% CI 1.08 to 4.04, *P* < 0.05) in PT and 2.14 (95% CI 1.17 to 3.92, *P* < 0.05) in CT. In model 2, age and systemic diseases were adjusted in PT. In PT, the OR of overall stigma was 1.89 (95% CI 1.02 to 3.53, *P* < 0.05) and the OR of enacted stigma was 1.99 (95% CI 1.02 to 3.89, *P* < 0.05). In addition, there was no significant association between IT and stigma.

## 4. Discussion

In the present study, the most important finding is that stigma, specifically enacted stigma, has an independent association with pain in TMD patients after adjustment for corresponding cofounders. Stigma, including overall stigma, enacted stigma, and felt stigma, was more associated with anxiety and depression and less related to jaw functional limitation of the patients with TMDs.

It is the first study to comprehensively investigate the association between stigma and TMD. Firstly, besides common demographic information of patients, we collected not only the data closely related to TMD, including pain level, psychological conditions, and adverse oral habits, but also other possibly relevant data such as education level and dental treatment experience. Secondly, apart from exploring the relationship between stigma and TMD, we further investigated the association of their corresponding subgroups. Thirdly, we also studied the correlations between stigma and other three scales. Based on the aspects above, it is believed that our findings are comprehensive and reliable.

The evidence base is unfortunately small and inadequate for stigma in TMDs, for the last study can date back to 1990, which mainly focused on the sources of stigma in temporomandibular pain and dysfunction syndrome (TMPDS) patients [20]. They believed that stigma is not the result of chronic pain itself but came from physicians or dentists when TMPDS patients sought for treatment [20], which was only partially consistent with our findings. Our finding coincided with Marbach et al. [20] on the results that enacted stigma, rather than felt stigma, was significantly related to PT and CT, indicating TMD patients mainly experienced stigma externally. In addition to the stigma from friends and family, other studies also noted that nurses, medicine physicians, and other medical workers could induce the stigma of painful patients when their pain did not have a clear basis in tissue pathology and was attributed to psychosocial factors [24–26]. Our study differed from the results of Marbach et al. [20] in that PT and CT, rather than IT, had significant association with the overall stigma and enacted stigma, demonstrating the chronic pain in TMD was associated with stigma. Consistent with our findings, Lies De Ruddere et al. [21] suggested that chronic nonmalignant pain made patients more sensitive to stigmatizing reactions of others. Hence, the reduction of chronic pain during the treatment of TMD may become more important in terms of stigma.

Our study found that stigma was associated with psychological conditions and revealed a moderate correlation between overall stigma and depression and anxiety, indicating that there may exist complex interactions between stigma and psychological conditions. Nowadays, more than 10% of global population lived with psychological problems, among which anxiety and depression were considered as the most common conditions [27]. Anxiety and depression can increase the patients' sensitivity and vulnerability to negative stereotypes [28] and may make TMD patients experience stigma more easily by suffering from negative mental health consequences. A recent study showed that more than one-third of patients with psychological problems experienced moderate levels of stigma and discrimination [29], indicating psychological burden may play a role in the emergence and aggravation of stigma. On the other hand, stigma in turn predisposed people from developing psychological problems [30, 31]. Boaz et al. [32] found that stigma often triggered social anxiety in bipolar disorder patients. Ali et al. [33] noted that stigma may contribute to poor psychological conditions in intellectual disabled patients. Aruta et al. [28] also found that people experiencing stigma tended to carry worse conditions of depression and anxiety and further proposed that stigma could positively predict depression and anxiety, indicating stigma could increase one's vulnerability to feel sad, hopeless, anxious, depressive, and other psychological emotions. Consequently, both elimination of stigma and psychological intervention need to be taken into account during the treatment of TMD.

Although our study showed the scores of JFLS-8 were significantly higher in patients with stigma than those without stigma, we found there was only a weak association between stigma and jaw functional limitation. This finding was the same in epilepsy [34] but contrary to stroke [35]. Zoppi et al. [34] found that stigma was poorly related to clinical aspects of epilepsy, while Lu et al. [35] suggested that severe stroke patients experienced more discrimination externally, raising both enacted stigma and felt stigma. The effect of dysfunction on stigma varies in different diseases. Our finding demonstrated that jaw function of TMD patients may play little role in stigma.

In our study, we found that patients with felt stigma mostly received a lower level of education, indicating that knowledge may be an important factor affecting patients' stigma. Our finding was consistent with public cognition that education cannot eradicate but can partially alleviate stigma caused by ignorance [36]. The level of education could affect people's mental health literacy, and higher education led to higher mental health literacy, which can mitigate the adverse effect of stigma on care seeking [37]. Another large randomized controlled trial also demonstrated that education on mental health literacy could reduce stigma by improving attitudes towards illness [38]. Hence, dentists should pay close attention to the stigmatized conditions of the patients with low educational attainment and reduce stigma during the management of TMD.

Besides, we also found that the patients with overall stigma presented worse oral habits, revealing that stigma may be associated with adverse oral habits. Although the direct association between stigma and oral habits was rarely explored, the relationship between adverse oral habits and psychological conditions of the patients has been widely investigated [39–43], which may explain the effect of adverse oral habits on stigma. Several studies indicated that patients with a higher incidence of bruxism suffered from a more severe depression and psychological stress [39, 42, 43]. It was also reported that tooth clenching could produce an over sevenfold increase in the BDI (Beck's Depression Inventory) level of the patients with painful TMD [41]. However, it still needs subsequent studies to investigate whether the influence of adverse oral habits on stigma is unidirectional or bidirectional.

Based on the recommendation of “Raise Awareness, Improve Education, and Reduce Stigma” in the monograph entitled “Temporomandibular Disorders: Priorities for Research and Care” published on National Academies Press [17], we investigated stigma in detail and found stigma was associated with pain in TMD patients, suggesting the importance of incorporating stigma management into routine TMD treatment. According to our findings, we could reduce stigma by monitoring the stigma conditions as well as other psychological conditions throughout the whole course of the TMD treatment. This is a preliminary study, and the research about intervention to reduce the stigma related to TMDs will be carried out afterwards.

Several limitations also existed in this study. Firstly, it was a cross-sectional study, and a longitudinal study will be needed to validate the association between stigma and TMD. Secondly, the study primarily focused on pain in the entire temporomandibular joint region. Pain in different regions, intensity, duration, and nature could be further investigated in detail. Thirdly, the age of the patients in this study ranged from 12 to 58 years including teenagers, adults, and elderly people. The scales adopted in our research were of general applicability regardless of the age difference. However, considering the age difference, it would be better to design different types of questionnaires and applied in corresponding groups. Fourthly, we evaluated stigma using the SSCI-8. A new stigma scale specific to TMDs focusing on enacted stigma could be designed and utilized in TMD patients in future studies. Besides, the stigma scale could be modified for use in different populations, especially for the teenage group. Despite these limitations, our research is a first step towards a profound understanding of pain-associated stigma in patients with TMDs.

## 5. Conclusion

Our results demonstrated that stigma, specifically enacted stigma, was correlated to pain from TMDs. Patients experiencing stigma were associated with psychological problems, indicating both elimination of stigma and psychological intervention need to be taken into account during the treatment of TMDs.

## Figures and Tables

**Table 1:** Sociodemographic and clinical characteristics of the patients.

Variable	Mean + SD or *n* (%)	Median (min–max)
Gender		
Male	56 (24.89%)	
Female	169 (75.11%)	

Age	27.30 ± 8.35	25 (12–58)
VAS current	1.35 ± 1.53	1 (0–8)
VAS highest	1.78 ± 1.85	1 (0–9)
GAD-7	3.77 ± 4.42	3 (0–21)
PHQ-9	3.67 ± 4.77	2 (0–27)
JFLS-8	11.41 ± 10.91	9 (0–43)
Overall stigma	2.02 ± 3.37	0 (0–16)
Felt stigma	1.04 ± 1.89	0 (0–8)
Enacted stigma	0.98 ± 1.86	0 (0–12)

Education		
Secondary school and below	37 (16.44%)	
Undergraduate or junior college	153 (68.00%)	
Postgraduate and above	35 (15.56%)	

Adverse oral habits		
No	48 (21.33%)	
Yes	177 (78.67%)	

Dental treatment		
No	57 (25.33%)	
Yes	168 (74.67%)	

Systemic diseases		
No	192 (85.33%)	
Yes	33 (14.67%)	

PT		
No	64 (28.44%)	
Yes	161 (71.56%)	

IT		
No	17 (7.56%)	
Yes	208 (92.44%)	

CT		
No	81 (36.00%)	
Yes	144 (64.00%)	

SD: standard deviation, VAS current: the current score of Visual Analogue Scale of Pain, VAS highest: the highest score of Visual Analogue Scale of Pain in the last one month, GAD-7: Generalized Anxiety Disorder 7-Item, PHQ-9: the Patient Health Questionnaire 9-item, JFLS-8: Jaw Functional Limitation Scale 8-item, PT: pain-related TMD, IT: intra-articular TMD, CT: combined TMD, and TMD: temporomandibular joint disorder.

**Table 2:** Effects of clinical characteristics and quantitative variables on overall stigma.

	Overall stigma		
	No	Yes	*P* value
*N*	121	104	

Gender			0.730
Male	29 (23.97%)	27 (25.96%)	
Female	92 (76.03%)	77 (74.04%)	

Age	26.70 ± 7.43	27.99 ± 9.28	0.249
VAS current	**1.14** ± **1.42**	**1.60** ± **1.62**	**0.025**
VAS highest	**1.55** ± **1.80**	**2.05** ± **1.89**	**0.046**
GAD-7	**2.21** ± **3.48**	**5.59** ± **4.71**	**<0.001**
PHQ-9	**2.07** ± **3.68**	**5.53** ± **5.22**	**<0.001**
JFLS-8	**8.61** ± **8.72**	**14.67** ± **12.26**	**<0.001**

Education			0.148
Secondary school and below	20 (16.53%)	17 (16.35%)	
Undergraduate or junior college	77 (63.64%)	76 (73.08%)	
Postgraduate and above	24 (19.83%)	11 (10.58%)	

Adverse oral habits			0.090
No	31 (25.62%)	17 (16.35%)	
Yes	90 (74.38%)	87 (83.65%)	

Dental treatment			0.915
No	31 (25.62%)	26 (25.00%)	
Yes	90 (74.38%)	78 (75.00%)	

Systemic diseases			0.509
No	105 (86.78%)	87 (83.65%)	
Yes	16 (13.22%)	17 (16.35%)	

PT			**0.025**
No	**42 (34.71%)**	**22 (21.15%)**	
Yes	**79 (65.29%)**	**82 (78.85%)**	

IT			0.148
No	12 (9.92%)	5 (4.81%)	
Yes	109 (90.08%)	99 (95.19%)	

CT			**0.004**
No	**54 (44.63%)**	**27 (25.96%)**	
Yes	**67 (55.37%)**	**77 (74.04%)**	

The chi-square test and Mann-Whitney *U* test are used. VAS current: the current score of Visual Analogue Scale of Pain, VAS highest: the highest score of Visual Analogue Scale of Pain in the last one month, GAD-7: Generalized Anxiety Disorder 7-item, PHQ-9: the Patient Health Questionnaire 9-item, JFLS-8: Jaw Functional Limitation Scale 8-item, PT: pain-related TMD, IT: intra-articular TMD, CT: combined TMD, and TMD: temporomandibular joint disorder. Statistically significant groups have been bolded.

**Table 3:** Effects of clinical characteristics and quantitative variables on felt stigma.

Variables	Felt stigma		
	No	Yes	*P* value
*N*	156	69	

Gender			0.345
Male	36 (23.08%)	20 (28.99%)	
Female	120 (76.92%)	49 (71.01%)	

Age	27.36 ± 8.07	27.16 ± 8.99	0.869
VAS current	1.28 ± 1.51	1.51 ± 1.57	0.309
VAS highest	1.74 ± 1.87	1.87 ± 1.83	0.639
GAD-7	**2.88** ± **3.81**	**5.80** ± **5.01**	**<0.001**
PHQ-9	**2.69** ± **4.32**	**5.87** ± **5.04**	**<0.001**
JFLS-8	**10.17** ± **9.96**	**14.23** ± **12.42**	**0.010**

Education			0.071
Secondary school and below	24 (15.38%)	13 (18.84%)	
Undergraduate or junior college	102 (65.38%)	51 (73.91%)	
Postgraduate and above	30 (19.23%)	5 (7.25%)	

Adverse oral habits			0.189
No	37 (23.72%)	11 (15.94%)	
Yes	119 (76.28%)	58 (84.06%)	

Dental treatment			0.613
No	38 (24.36%)	19 (27.54%)	
Yes	118 (75.64%)	50 (72.46%)	

Systemic diseases			0.961
No	133 (85.26%)	59 (85.51%)	
Yes	23 (14.74%)	10 (14.49%)	

PT			0.602
No	46 (29.49%)	18 (26.09%)	
Yes	110 (70.51%)	51 (73.91%)	

IT			0.079
No	15 (9.62%)	2 (2.90%)	
Yes	141 (90.38%)	67 (97.10%)	

CT			0.145
No	61 (39.10%)	20 (28.99%)	
Yes	95 (60.90%)	49 (71.01%)	

The chi-square test and Mann-Whitney *U* test are used. VAS current: the current score of Visual Analogue Scale of Pain, VAS highest: the highest score of Visual Analogue Scale of Pain in the last one month, GAD-7: Generalized Anxiety Disorder 7-item, PHQ-9: the Patient Health Questionnaire 9-item, JFLS-8: Jaw Functional Limitation Scale 8-item, PT: pain-related TMD, IT: intra-articular TMD, CT: combined TMD, and TMD: temporomandibular joint disorder. Statistically significant groups have been bolded.

**Table 4:** Effects of clinical characteristics and quantitative variables on enacted stigma.

Variables	Enacted stigma		
	No	Yes	*P* value
*N*	140	85	

Gender			0.788
Male	34 (24.29%)	22 (25.88%)	
Female	106 (75.71%)	63 (74.12%)	

Age	26.45 ± 7.21	28.69 ± 9.83	0.050
VAS current	**1.14** ± **1.38**	**1.69** ± **1.70**	**0.008**
VAS highest	**1.56** ± **1.79**	**2.14** ± **1.92**	**0.023**
GAD-7	**2.64** ± **3.92**	**5.64** ± **4.57**	**<0.001**
PHQ-9	**2.57** ± **4.11**	**5.47** ± **5.25**	**<0.001**
JFLS-8	**8.97** ± **8.95**	**15.44** ± **12.59**	**<0.001**

Education			0.394
Secondary school and below	24 (17.14%)	13 (15.29%)	
Undergraduate or junior college	91 (65.00%)	62 (72.94%)	
Postgraduate and above	25 (17.86%)	10 (11.76%)	

Adverse oral habits			0.165
No	34 (24.29%)	14 (16.47%)	
Yes	106 (75.71%)	71 (83.53%)	

Dental treatment			0.264
No	39 (27.86%)	18 (21.18%)	
Yes	101 (72.14%)	67 (78.82%)	

Systemic diseases			0.170
No	123 (87.86%)	69 (81.18%)	
Yes	17 (12.14%)	16 (18.82%)	

PT			**0.013**
No	**48 (34.29%)**	**16 (18.82%)**	
Yes	**92 (65.71%)**	**69 (81.18%)**	

IT			0.459
No	12 (8.57%)	5 (5.88%)	
Yes	128 (91.43%)	80 (94.12%)	

CT			**0.006**
No	**60 (42.86%)**	**21 (24.71%)**	
Yes	**80 (57.14%)**	**64 (75.29%)**	

The chi-square test and Mann-Whitney *U* test are used. VAS current: the current score of Visual Analogue Scale of Pain, VAS highest: the highest score of Visual Analogue Scale of Pain in the last one month, GAD-7: Generalized Anxiety Disorder 7-Item, PHQ-9: the Patient Health Questionnaire 9-item, JFLS-8: Jaw Functional Limitation Scale 8-item, PT: pain-related TMD, IT: intra-articular TMD, CT: combined TMD, and TMD: temporomandibular joint disorder. Statistically significant groups have been bolded.

**Table 5:** Crude association of PT, IT, and CT with gender, age, education level, systemic diseases, dental treatment, adverse oral habits, overall stigma, felt stigma, and enacted stigma.

	PT	IT	CT
Gender			
Male	Reference	Reference	Reference
Female	1.42 (0.74, 2.71)	1.28 (0.43, 3.82)	1.47 (0.79, 2.73)

Age	**1.08 (1.03, 1.13) ** ^*∗*^	0.98 (0.93, 1.03)	**1.05 (1.01, 1.09) ** ^*∗*^
Education			
Secondary school and below	Reference	Reference	Reference
Undergraduate or junior college	1.15 (0.53, 2.49)	0.00 (0.00, Inf)	0.79 (0.37, 1.68)
Postgraduate and above	1.92 (0.65, 5.64)	0.00 (0.00, Inf)	1.05 (0.39, 2.82)

Systemic diseases			
No	Reference	Reference	Reference
Yes	**4.66 (1.37, 15.85) ** ^*∗*^	0.37 (0.12, 1.14)	1.92 (0.82, 4.48)

Dental treatment			
No	Reference	Reference	Reference
Yes	1.70 (0.89, 3.22)	0.61 (0.17, 2.21)	1.42 (0.77, 2.62)

Adverse oral habits			
No	Reference	Reference	Reference
Yes	1.05 (0.52, 2.11)	2.16 (0.75, 6.17)	1.36 (0.71, 2.61)

Overall stigma			
No	Reference	Reference	Reference
Yes	**1.98 (1.09, 3.62) ** ^*∗*^	2.18 (0.74, 6.41)	**2.30 (1.30, 4.05) ** ^*∗*^

Felt stigma			
No	Reference	Reference	Reference
Yes	1.18 (0.63, 2.24)	3.56 (0.79, 16.03)	1.57 (0.85, 2.90)

Enacted stigma			
No	Reference	Reference	Reference
Yes	**2.25 (1.18, 4.29) ** ^*∗*^	1.50 (0.51, 4.42)	**2.29 (1.26, 4.15) ** ^*∗*^

PT: pain-related TMD, IT: intra-articular TMD, CT: combined TMD, and TMD: temporomandibular joint disorder. Values are regression coefficients (95% confidence interval) from univariate regression models and reflect differences in PT, IT, and CT per unit change of each covariate and for different categories of each covariate as compared to the reference group. ^*∗*^*P* value <0.05. Statistically significant groups have been bolded.

**Table 6:** Multivariable linear regression analyses for PT, IT, and CT in overall stigma, felt stigma, and enacted stigma.

	Model 1		Model 2	
	OR, 95% CI (low to high)	*P* value	OR, 95% CI (low to high)	*P* value
PT				
Overall stigma	1.93 (1.04, 3.58)^1^	**0.0361**	1.89 (1.02, 3.53)^2^	**0.0443**
Felt stigma	1.24 (0.64, 2.38)^1^	0.5252	1.29 (0.66, 2.52)^3^	0.4612
Enacted stigma	2.09 (1.08, 4.04)^1^	**0.0288**	1.99 (1.02, 3.89)^2^	**0.0426**

IT				
Overall stigma	2.31 (0.78, 6.85)^4^	0.1330	—	—
Felt stigma	3.60 (0.80, 16.32)^4^	0.0961	—	—
Enacted stigma	1.51 (0.51, 4.44)^5^	0.4570	1.63 (0.53, 4.97)^6^	0.3912

CT				
Overall stigma	2.24 (1.26, 3.98)^1^	**0.0058**	—	—
Felt stigma	1.63 (0.87, 3.03)^1^	0.1256	—	—
Enacted stigma	2.14 (1.17, 3.92)^1^	**0.0133**	—	—

PT: pain-related TMD, IT: intra-articular TMD, CT: combined TMD, TMD: temporomandibular joint disorder. ^1^ means adjusted for age. ^2^ means adjusted for age and systemic diseases. ^3^ means adjusted for age, sex, education, systemic diseases, and dental treatment. ^4^ means adjusted for systemic diseases. ^5^ means adjusted for sex. ^6^ means adjusted for sex, age, systemic diseases, and adverse oral habits. Statistically significant groups have been bolded.

## Data Availability

Data are available on request.
